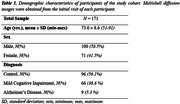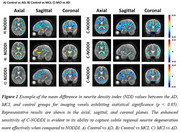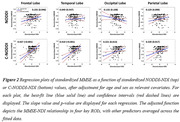# Widespread neurite degeneration in mild cognitive impairment and Alzheimer’s disease revealed using advanced diffusion MRI

**DOI:** 10.1002/alz.093754

**Published:** 2025-01-09

**Authors:** John P Laporte, Zhaoyuan Gong, Alex Guo, Jonghyun Bae, Mary E Faulkner, Mustapha Bouhrara

**Affiliations:** ^1^ National Institute on Aging, Baltimore, MD USA; ^2^ Laboratory of Clinical Investigation, National Institute on Aging, Intramural Research Program, Baltimore, MD USA

## Abstract

**Background:**

Neurite degeneration is increasingly suspected to represent a causal feature of mild cognitive impairment (MCI) and Alzheimer’s disease (AD). Therefore, sensitive and specific imaging biomarkers of neuronal degeneration are needed to elucidate the mechanisms underlying cognitive impairment in MCI and AD. However, the recently developed Neurite Orientation Dispersion and Density Imaging (NODDI) MRI technique, used to measure the neurite density index (NDI), has some limitations. To address these, we have introduced the constrained NODDI (C‐NODDI) analysis providing more accurate determination of NDI (PMC10435293). Here, we investigated neurite degeneration in MCI and AD using C‐NODDI and compared the results to those derived using NODDI.

**Methods:**

We obtained Multi‐shell diffusion MR images from the ADNI study (Table 1) and calculated NDI using NODDI or C‐NODDI. We conducted a voxelwise two‐sample t‐test to compare NDI of control vs. AD, control vs. MCI, and MCI vs. AD groups. Further, we assessed the regional associations between NDI and overall cognition as measured using the Mini‐Mental State Examination (MMSE) score (dependent variable), controlling for age and sex. All continuous variables were Z‐scored.

**Results:**

The AD group exhibited significantly lower NDI values as compared to control, with C‐NODDI exhibiting greater sensitivity in capturing regional NDI variations as compared to NODDI (Figure.1). Further, unlike NODDI, C‐NODDI was capable of detecting subtitle differences in NDI between control and MCI and between MCI and AD (Figure.1). Interestingly, NODDI exhibited higher NDI values in MCI or AD as compared to control in several brain regions (Figure.1), a physiologically implausible result that C‐NODDI corrected. Additionally, the associations between MMSE and NDI derived using C‐NODDI were significant in several white matter regions, with higher NDI values corresponding to expected higher MMSE scores (Figure.2). NODDI has failed to capture this anticipated association.

**Conclusions:**

This study provides evidence of widespread neuronal degeneration in MCI and AD. Importantly, our results highlight the superior sensitivity of C‐NODDI analysis in detecting these differences in neurite density as compared to NODDI, making it a potent MRI method for clinical investigations and trials studying neuronal degeneration in aging and age‐related diseases.